# Salivary Fistula Cured by the Galvano-Cautery

**Published:** 1887-07

**Authors:** 


### Salivary Fistula Cured by the Galvano-Cautery.

In the *Glasgow Med. Jour.*, February, 1887, Professor George Buchanan records an example of salivary fistula cured by the galvano-cautery. A lady suffered from an enormous carbuncle situated on the left side of the neck, below the ear, extending to the cheek over the parotid gland. A free incision was made, and the parts almost completely cicatrized, but below the lobule of the ear a little sinus remained, from which a small quantity of semi-purulent watery fluid exuded. For thirteen years the patient had nothing done; every time mastication was performed some drops of saliva issued from the fistula below the ear. The author saw her in October, 1886, and recommended cauterization of the mouth of the fistula. A platinum wire loop was shortened and pressed together, so that the two sides of the loop were in contact, and together were not thicker than a small probe. The wire was placed on the exact spot of the fistula before it was heated, and held firmly there whilst heated by the electric current. An eschar, the size of two pins' heads, was produced by the wire, and a fortnight later the sinus was perfectly healed.

